# Performance Improvement of Substrate Integrated Cavity Fed Dipole Array Antenna Using ENZ Metamaterial for 5G Applications

**DOI:** 10.3390/s22010125

**Published:** 2021-12-25

**Authors:** Shaza El-Nady, Rania R. Elsharkawy, Asmaa I. Afifi, Anwer S. Abd El-Hameed

**Affiliations:** Electronics Research Institute, Microstrip Circuits Joseph Tito St, Huckstep, El Nozha, Cairo 11843, Egypt; raniarefaat85@eri.sci.eg (R.R.E.); asmaa.afifi@ejust.edu.eg (A.I.A.)

**Keywords:** array, dipole, ENZ, mm-wave, reflection coefficient, SIC

## Abstract

This paper exhibits a high-gain, low-profile dipole antenna array (DAA) for 5G applications. The dipole element has a semi-triangular shape to realize a simple input impedance regime. To reduce the overall antenna size, a substrate integrated cavity (SIC) is adopted as a power splitter feeding network. The transition between the SIC and the antenna element is achieved by a grounded coplanar waveguide (GCPW) to increase the degree of freedom of impedance matching. Epsilon-near-zero (ENZ) metamaterial technique is exploited for gain enhancement. The ENZ metamaterial unit cells of meander shape are placed in front of each dipole perpendicularly to guide the radiated power into the broadside direction. The prospective antenna has an overall size of 2.58 $\lambda_{g}^{3}$ and operates from 28.5 GHz up to 30.5 GHz. The gain is improved by 5 dB compared to that of the antenna without ENZ unit cells, reaching 11 dBi at the center frequency of 29.5 GHz. Measured and simulated results show a reasonable agreement.

## 1. Introduction

Nowadays, the demand for millimeter-wave (mm) wireless systems has increased due to their gigabyte data rates. Consequently, several teams have been adopted by the International Telecommunication Union (ITU) to accomplish all 5G standards before 2020. The ITU assigned the frequency bands of the recent mobile generation (5G) between 24 GHz and 86 GHz [1]. Although the 5G bands are not completely established, various bands are candidates [2]. The frequency bands such as sub 3 GHz, sub 6 GHz, and sub-millimeter bands are endorsed for 5G applications. Nonetheless, the millimeter bands are highly recommended by academics than the low frequencies. The reason is that the low-frequency spectrum is congested and occupied by many applications. Several pieces of research have been reported considering the frequency band from 28 GHz to 38 GHz for 5G applications [3,4].

The antenna is an indispensable element for any wireless communication system. To introduce a successful antenna at mm-wave bands, important factors should be considered; the high attenuation due to atmospheric absorption (AA) and the free space loss (FSL). To overcome these challenges, a high-gain antenna is highly recommended. Besides, the antenna size and operating bandwidth should be taken into account. To satisfy most of these requirements, different techniques have been investigated, including antenna arrays based on planar waveguides [5,6], reflectarrays [7], substrate integrated waveguides (SIWs) [8,9], and beam-forming circuits [10].

The most straightforward way to enhance the antenna gain is to construct arrays of multiple elements [5]. A modified compact broadband antipodal Vivaldi antenna (AVA) array was proposed for 5G applications to provide a maximum gain of 11.32 dBi [11]. However, the feeding network is the major drawback due to its high complexity and large size.

The other method for gain improvement is the utilization of metamaterials. Metamaterials have a conspicuous interest due to their phenomenal electromagnetic properties, which do not exist in common materials, like double negative (DNG) refractive index metamaterials [12], mu-near-zero metamaterials (MNZ) [13], and epsilon-near-zero (ENZ) metamaterials [14]. In an ENZ metamaterial, the phase velocity of a plane wave tends to infinity resulting in wavelengths tending to infinity, which means no phase variations in such material. So, if a radiator is implanted into an ideal ENZ metamaterial, the beams emerging from distinct points of the ENZ surface are in phase. This property enables the ENZ metamaterial to be an excellent candidate for antenna radiation enhancement [14]. Three pairs of metamaterial arrays were utilized to improve the gain of the dual-beam end-fire bowtie antenna to 7.4 dBi at 26 GHz [15]. A modified AVA with metamaterial and corrugation enhanced the gain from 5 to 9.53 dBi over 24.77 to 34.52 GHz [16]. However, the antenna array leads to large sizes, while the ENZ metamaterial technique leads unsatisfactory gain.

In addition, various techniques were utilized to improve the antenna gain [17,18,19,20]. Higher mode excitations in dielectric resonator antennas (DRAs) were used to improve the antenna gain to 10.4 dBi at 16.5 GHz [17]. Four parallel transverse slots were etched at the edges of the hexagonal SIC to generate a higher resonance mode of quasi-TM310 to achieve a maximum gain of 11 dBi at 30.5 GHz [18]. In [19], the gain has been increased based on stacking different dielectric resonators (DR) with semi-circular slots etched on the left and right sides of the radiator. Rectangular, circular, and zigzag-shaped defected ground structures were etched beneath the radiating elements to enhance the antenna gain to 8.3 dBi at 29.6 GHz [20].

Substrate Integrated Waveguide (SIW) is a promising technology, which yields a compact size and low cost of planar or non-planar microwave and mm-wave circuits with high performance [21]. Several techniques and circuits have been studied and realized using SIWs, like Tee structures, oscillators, six-port junctions, and waveguide slot array antennas [22]. The high-performance transition between planar transmission lines and SIWs is the key to build various compact passive circuits. In [18,23], power splitting and an improved impedance bandwidth were realized using a substrate integrated cavity (SIC) technique, reducing the antenna size.

In this paper, a compact structure of a two-element antenna array combined with ENZ metamaterials for gain enhancement is presented. The suggested antenna is fed by an SIC power splitter in order to reduce the size of the structure. An antenna gain of 11 dBi is achieved across the operating bandwidth starting from 28.5 to 30.5 GHz, which is superb for future mm-wave applications. The introduced antenna has a small size of 8.5 m × 8.5 m × 5.5 m, and it exhibits good impedance matching, radiation patterns, and gain across the whole operating band. Through this paper, simulations have been carried out with the CST-MWS and verified with measurements.

This paper is arranged as follows. In Section 2, the analysis of the circular SIC-based antenna is presented. Section 3 gives a discussion of the detailed design of the ENZ cells and the effect of adding these cells to the proposed antenna. Section 4 summarizes the experimental results.

## 2. Circular SIC-Antenna Design

### 2.1. Circular SIC Power Splitter Design

**Figure 1:**
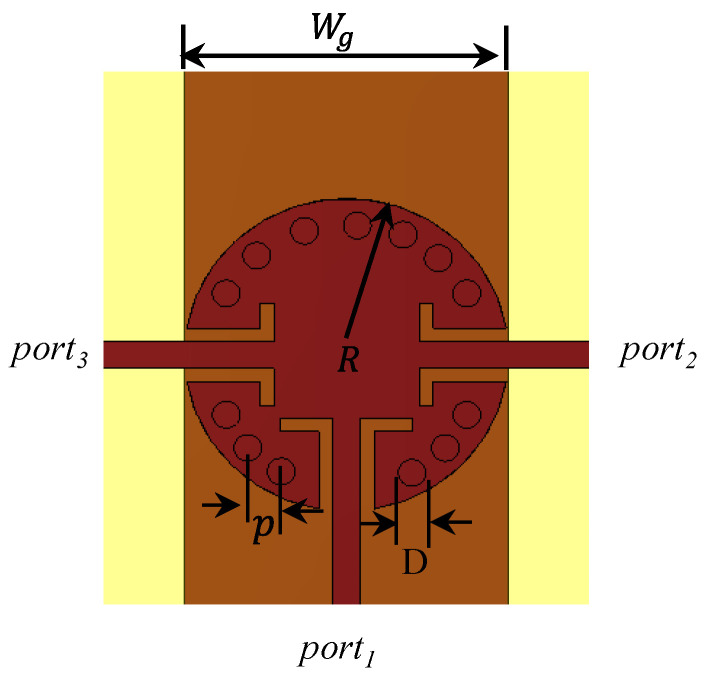
Power splitter configuration.

In this section, a design of a compact one-input/two-output ports power splitter is presented to be used as a feeding network for the prospective antenna array. A circular SIC with radius R=2.34 mm was elected to do this function, as shown in Figure 1, due to its compact size and wide bandwidth. The basic idea of this splitter originally comes from connecting the top and bottom layers by metalized via holes to eliminate unwanted modes, which cause high loss through the utilization of the shorting technique [24]. The metallic via position was carefully optimized to avoid resonance from the dielectric-loaded waveguide modes in the operating bandwidth. Furthermore, the via holes design satisfies the condition of D/p≥0.5 to reduce their losses, where *D* is the diameter of the via, and p is the distance between the two centers of adjacent vias. To avoid the radiation losses of the wall of the metal via holes, the spacing (p) should be less than half the guided wavelength at the highest frequency of interest [25]. In this design, an angle of 22.5 degrees between each two adjacent metal via holes in the SIC was considered. A genetic algorithm of CST-MWS software was utilized to obtain the optimum parameters of the proposed design. The optimum values of p, and D are 0.7 mm, and 0.4 mm, respectively. The optimized width of the ground plane is $W_{g}=3.97\;\mathrm{mm}$. Unlike the conventional power splitters that have quarter wavelength arms [26,27], the operating frequency of the cavity is determined by the dimensions of the cavity as demonstrated in Equation (1) [25]:

(1) $$f_{010}=\frac{c}{2\pi \sqrt{\mu_{r}\varepsilon_{r}}}\frac{B_{01}}{R}$$

where c is the light speed in free space,$\;\mu_{r\;}\mathrm{and}\;\varepsilon_{r}$ are the relative permeability and permittivity of the dielectric, respectively, R is the cavity radius, $B_{01}$ is the Bessel function.

**Figure 2:**
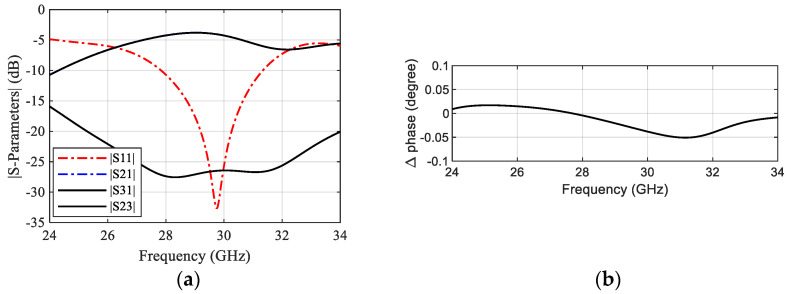
Power splitter performance. (**a**) S-parameters of the circular SIC power splitter. (**b**) Phase variations.

Figure 2a shows the simulated S-parameters of the power splitter. It can be observed that the power splitter achieves an impedance matching with $\left|S_{11}\right|$ below −10 dB from 27.8 to 31 GHz with a center of resonance at 29.5 GHz. The transmission coefficients $\left|S_{21}\right|$ and $\left|S_{31}\right|$ are identical across the entire bandwidth, indicating that the input power can be divided equally between the two outputs. A very low insertion loss of 0.2 dB and a good isolation of more than 25 dB are attained at the center frequency. Furthermore, the phase characteristic variation between the two output ports is less than $0.1^{o}$ indicating excellent performance in the scenario of integration with the antenna, as shown in Figure 2b. The GCPW is used as a transition stage between the microstrip line and the SIC. So, the input power could be divided between the two symmetric outputs.

### 2.2. Single Dipole Antenna Element

**Figure 3:**
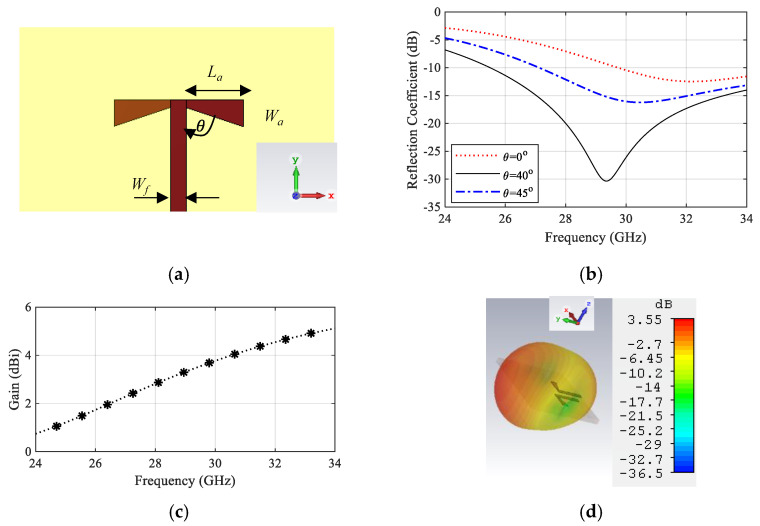
Geometry of the semi−triangular DA and its results. (**a**) Antenna configuration. (**b**) Antenna reflection coefficient. (**c**) Antenna gain versus frequency. (**d**) Antenna 3D radiation pattern at 29.5 GHz.

Figure 3 shows the geometry of the suggested DA. The antenna structure was chosen with a semi-triangular shape to control its input impedance easily by changing the angle θ. The substrate used in this design is Rogers 6035 with a dielectric constant of 3.6, a thickness of 0.508 mm, and a loss tangent of 0.0013. A 50-Ω microstrip line with a width $W_{f}=0.4\;\mathrm{mm}$ is used to feed the DA. Figure 3b shows the reflection coefficient for different angles θ demonstrating that θ can control the impedance matching efficiently. The initial value of the radiating element length of 2 × $L_{a}$ is set to the half-guided wavelength corresponding to 29.5 GHz, while the width $W_{a}$ and angle θ are calculated based on the quasi-static method presented in [28]. The optimized radiating element length $L_{a}$, the width $W_{a}$ and the angle θ are 1.4 mm, 0.7 mm, and 40°, respectively. The final DA covers a bandwidth starting from 26 GHz to more than 34 GHz with resonance at 29.5 GHz, similar to that of the SIC power splitter. The simulated gain of the DA shows a value of about 3.55 dBi at 29.5 GHz, as displayed in Figure 3c. The 3D radiation pattern of the antenna is illustrated at 29.5 GHz in Figure 3d.

### 2.3. Dipole Antenna Array

**Figure 4:**
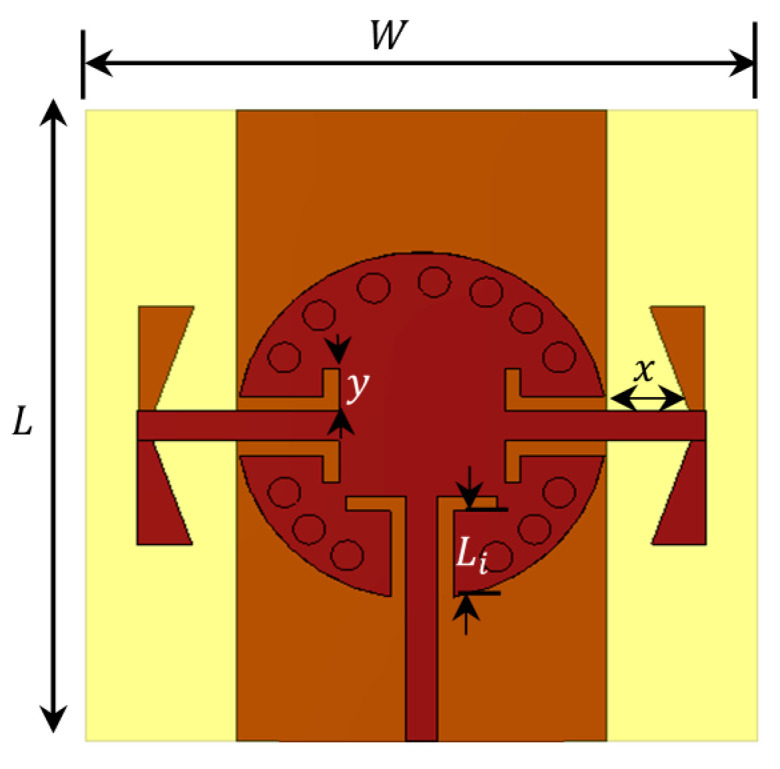
Configuration of the proposed antenna array.

**Figure 5:**
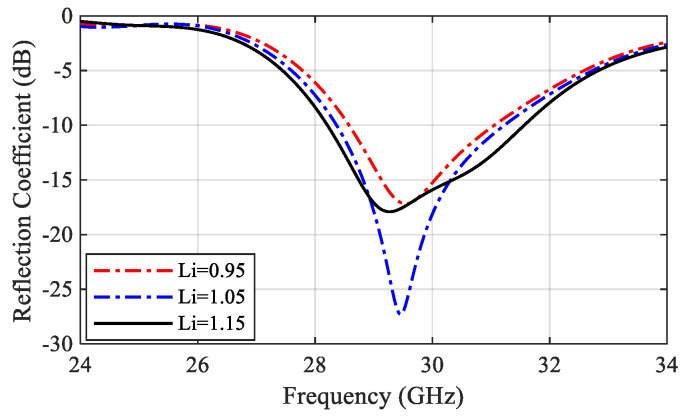
Effect of the CPW length $L_{i}$ on the antenna array reflection coefficient.

**Figure 6:**
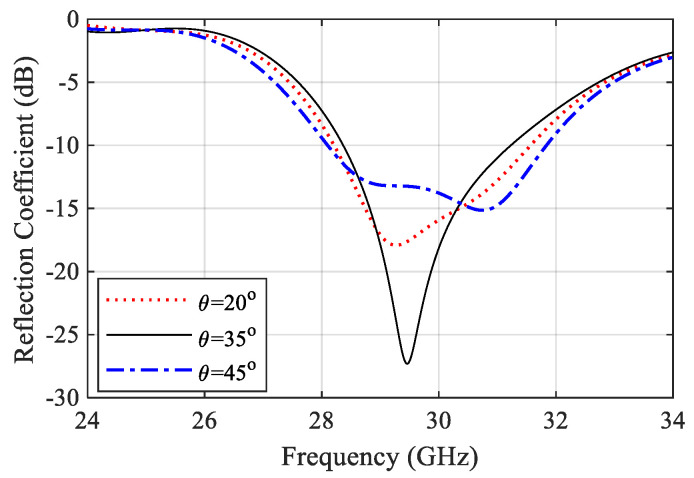
Effect of the dipole angle on the antenna array reflection coefficient.

Referred to the designed power splitter in Section 2.1, the DA elements were connected to the power splitter output ports. The final antenna array configuration is presented in Figure 4. The impedance matching performance of the antenna array is mainly influenced by the length of the GCPW transformers between the SIC and the microstrip line $L_{i}$ and the radiating dipole corner angle θ. To demonstrate the effect of these parameters, a parametric sweep was carried out, as shown in Figure 5 and Figure 6. The antenna impedance matching is obviously affected by both angle θ and length x, showing the best matching from 28.5 GHz to 31.5 GHz. The optimum antenna dimensions are listed in Table 1.

## 3. Gain Enhancement with ENZ Unit Cell

### 3.1. Unit Cell Design

**Figure 7:**
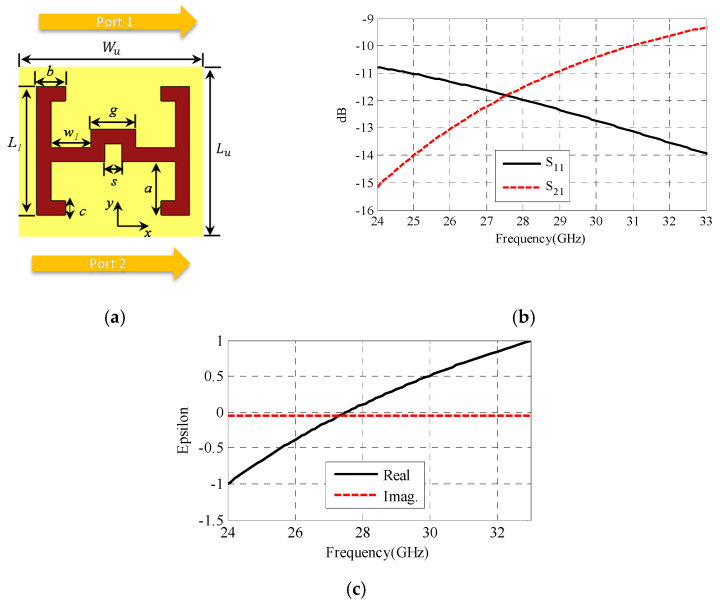
ENZ metamaterial unit cell and results. (**a**) Geometry of the unit cell. (**b**) S−parameters of the unit cell versus frequency. (**c**) Real and imaginary unit cell permittivity versus frequency.

In this section, the design of the ENZ unit cell is presented. The ENZ unit cell structure was modified from that in [29], as shown in Figure 7a. The unit cell was optimized to fit the required band from 28.5 GHz to 30.5 GHz. These unit cells were analyzed on the same dielectric substrate used for SIC with a periodicity smaller than one wavelength at 29.5 GHz. The dimensions of the ENZ unit cell are presented in Table 2. Figure 7b shows the transmission and reflection coefficients of the unit cell used to retrieve the corresponding effective permittivity [30]. Figure 7c shows the obtained effective permittivity, which is close to zero at the required band.

**Figure 8:**
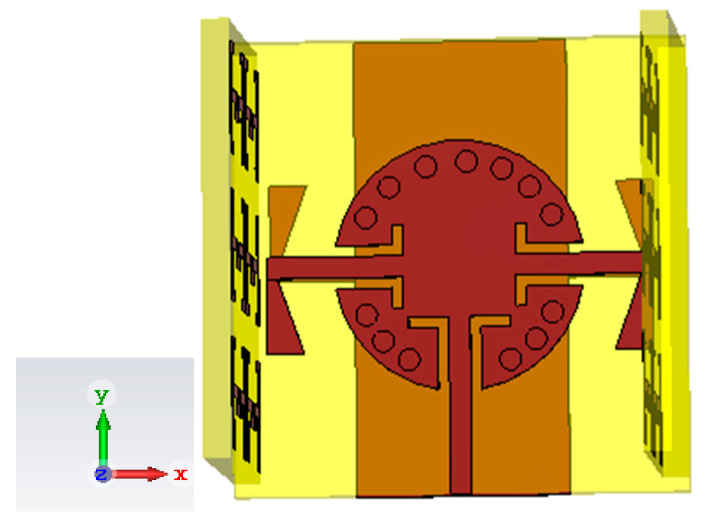
Antenna array configuration, including ENZ metamaterial slabs.

A lattice of 3 × 2 ENZ metamaterial unit cells was configured on a substrate slab of dimensions 5.5 m × 8 m. This arrangement was chosen to fit the length of the SIC cavity towards the z-axis as shown in Figure 8.

### 3.2. Circular SIC Antenna with ENZ Unit Cell Design

**Figure 9:**
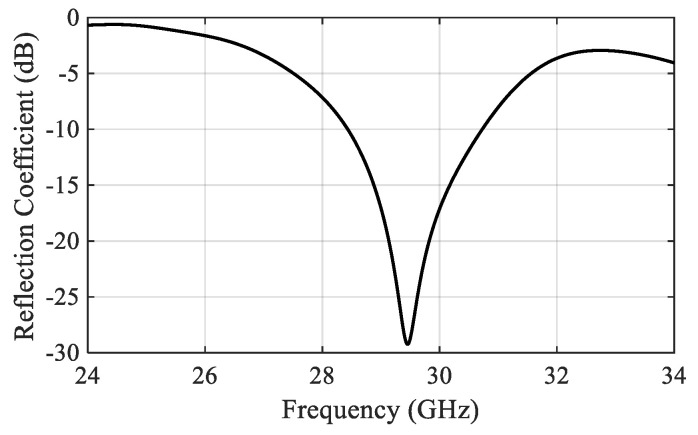
Reflection coefficient versus frequency of the antenna array, including ENZ metamaterial slabs.

**Figure 10:**
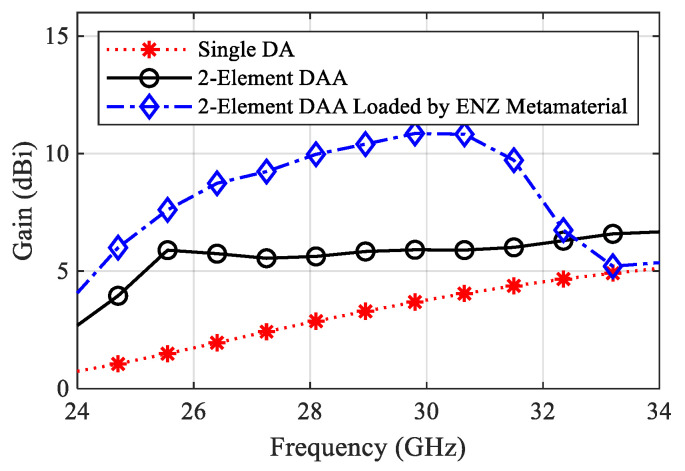
Gain versus frequency for all update steps.

**Figure 11:**
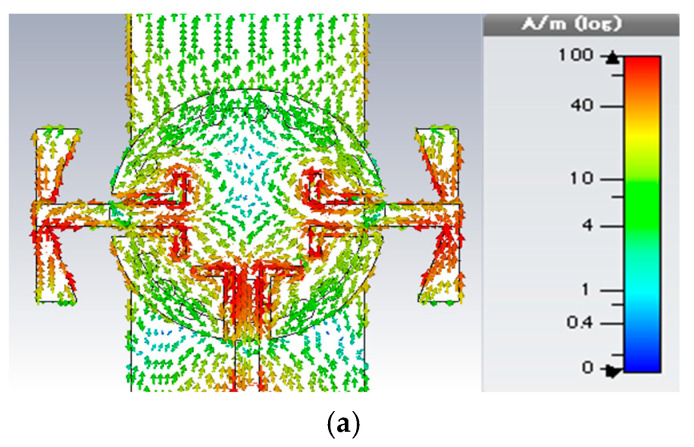

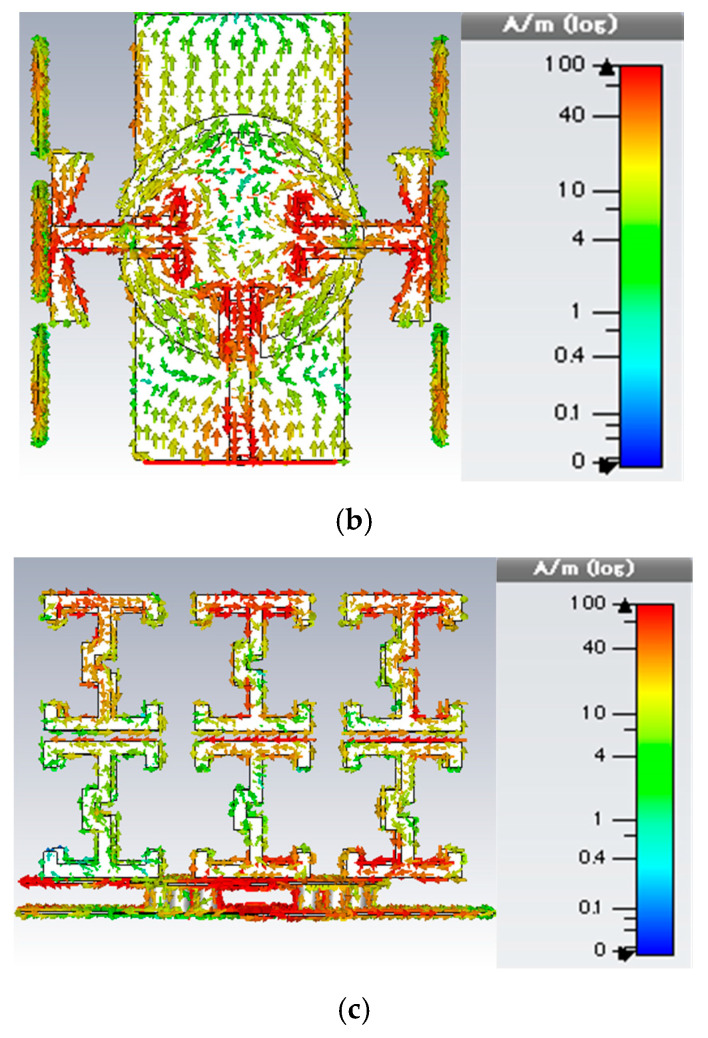
Current distribution. (**a**) Current distribution on an antenna without ENZ unit cells (**b**) Current distribution on the antenna with ENZ unit cells (**c**) Current distribution on the ENZ unit cells.

The suggested ENZ metamaterial lattice is used to enhance the broadside gain and to reduce the cross-section radiation of the proposed antenna. The bandwidth of the antenna is not affected so much by the presence of the ENZ unit cells, and the reflection coefficient shows very good matching across the bandwidth starting from 28.5 GHz to 30.5 GHz, as shown in Figure 9. The gain comparison for all update steps is demonstrated in Figure 10. The effect of the ENZ metamaterial is significant on the antenna gain over the whole bandwidth. The average gain is improved to 10.17 dBi, and the peak gain jumps to 11 dBi at 30 GHz. In other words, a 50% gain enhancement is achieved compared with the case of no ENZ unit cells. For further interpretation, the surface current distribution is illustrated in Figure 11. It is clear that the surface currents without ENZ unit cells are symmetrically distributed in the same direction on the array elements, which increases the radiation in the broadside direction and decreases it in the cross direction. After employing the ENZ metamaterial unit cells, the current is induced to the unit cells and distributed symmetrically, as shown in Figure 11b,c contributing to the traveling of the electromagnetic field uniformly along the z-direction, which improves the antenna gain.

## 4. Measurement Results

**Figure 12:**
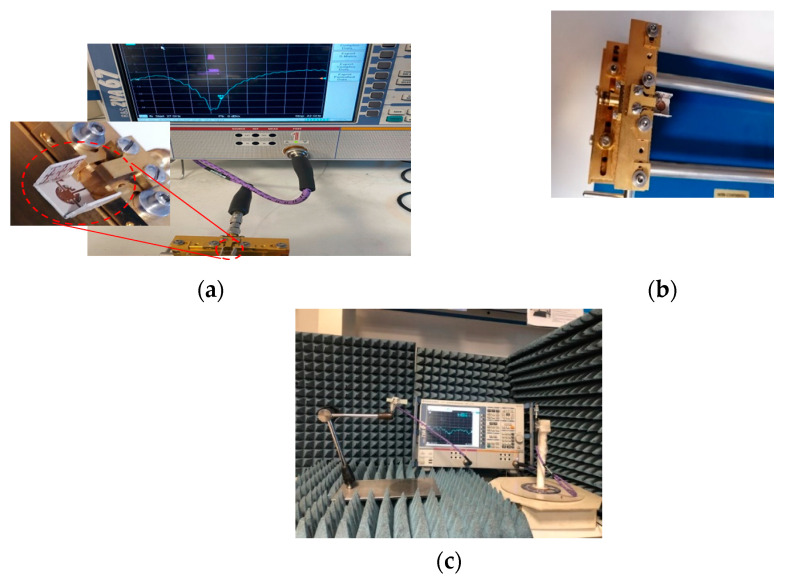
Fabricated antenna prototype. (**a**) Measurement setup of scattering parameters (**b**) Photo of the proposed antenna connected to the test fixture (**c**) Measurement setup of radiation patterns.

**Figure 13:**
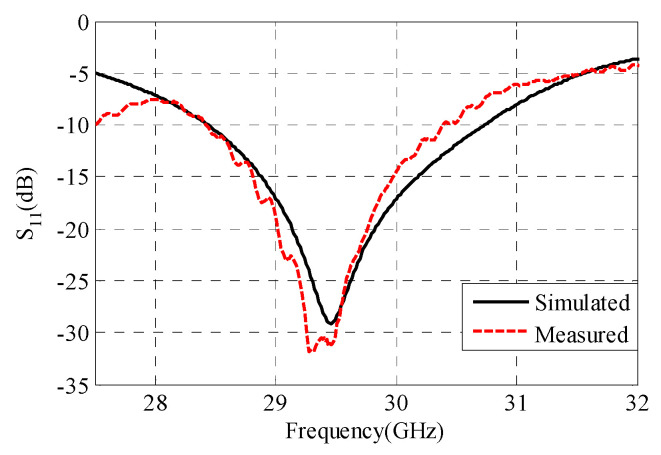
Measured and simulated reflection coefficients versus frequency.

**Figure 14:**
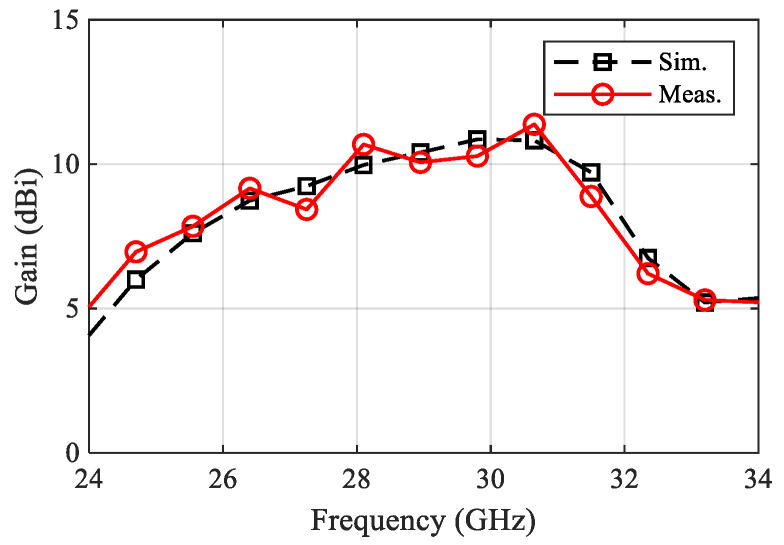
Measured and simulated gain versus frequency.

**Figure 15:**
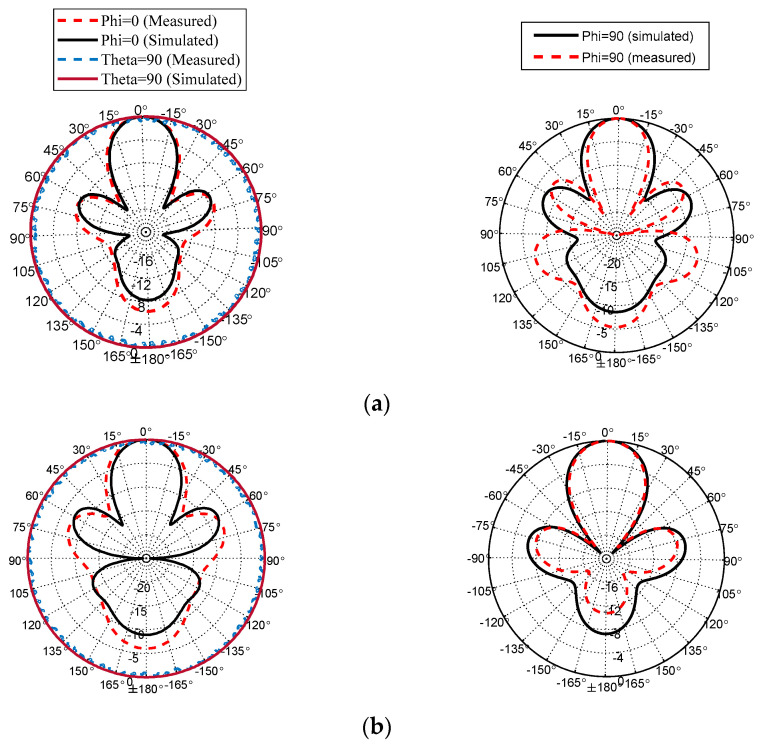

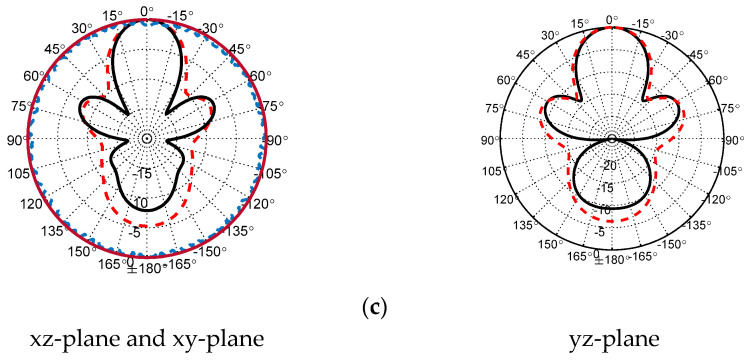
Antenna radiation patterns. (**a**) At 28.5 GHz . (**b**) At 29.5 GHz. (**c**) At 30.5 GHz.

A fabricated prototype of the proposed antenna is illustrated in Figure 12. An adhesive material was used to support the ENZ substrate to simplify the assembling stage. Testing was carried out by Vector Network Analyzer ZVA and a universal substrate test fixture of model number WK-3001 as shown in Figure 12b. The radiation pattern measurement setup is presented in Figure 12c. An mm-wave standard horn antenna was utilized as a transmitter, while the proposed antenna represented the receiver side. The distance between the transmitter and receiver antennas was kept to be more than one wavelength. To achieve accurate gain measurements, both antennas should be illuminated by a uniform plane wave. This means that a region free from reflections and unwanted signals should be considered. In practice, mm-wave absorbing materials were placed around the measurement system to eliminate any undesired interference. As shown in Figure 13, the input impedance is well-matched over the desired frequency band. However, the measured input impedance bandwidth is slightly narrower than the simulated one. The simulated impedance bandwidth starts from 28.5 GHz to 30.7 GHz, while the measured one ranges from 28.5 GHz to 30.5 GHz. The small difference between the measured and simulated reflection coefficient may be attributed to several factors including fabrication tolerance and effect of test fixture, which was not taken into account during the simulation process. The antenna radiation characteristics were measured using the gain-transfer method [31], showing a maximum gain of 11 dBi at 29.5 GHz with good agreement between measured and simulated curves, as shown in Figure 14. Figure 15 gives antenna radiation patterns in the three xz, xy, and yz planes at 28.5 GHz, 29.5 GHz, and 30.5 GHz, respectively. The measured results show some discrepancies due to fabrication tolerance and existence of the VNA and the metallic antenna holders inside the chamber. However, the measured and simulated results show quasi-broadside distribution. Generally, these measured results confirm the ability of the proposed structure to be a good candidate for mm-wave 5G applications. To show the advantage of the proposed antenna over the similar state-of-the-art work, Table 3 presents a comparison in terms of size and gain. It is clear that the proposed antenna has a distinctively high gain for a relatively compact size. Although the authors of [15,19,24] used smaller sizes, their designs suffer from either lower gain or complex structure.

## 5. Conclusions

The main challenges of size reduction and gain improvement have been treated for 5G applications in this paper. Two techniques have been exploited to accomplish this purpose. The first one depends on the idea of the SIC power splitter to feed two antenna elements with equal power, which simply reduces the antenna size, unlike complicated array feed networks. The other technique is the ENZ metamaterial which enhances the antenna gain with up to 5 dBi for the desired frequency band. This design reduces the manufacturing cost and simplifies the fabrication process. The prospective structure works in the range from 28.5 GHz up to 30.5 GHz, with an overall size of 2.58 $\lambda_{g}^{3}$. To validate the above-mentioned design, the antenna was fabricated, and the measured results agree well with the simulation results.

## Figures and Tables

**Table 1:** Optimum dimensions of the DAA in mm.

*W*	*L*	*Y*	*L_i_*	*x*
8.5	8.5	0.57	1.05	1.09

**Table 2:** Optimum dimensions of the ENZ metamaterial unit cell in mm.

$L_{u}\;$	$W_{u}\;$	*L*_1_	*W*_1_	*g*	*S*	*b*	*c*	*a*
2.5	2.5	2.04	0.629	0.716	0.26	0.456	0.228	0.85

**Table 3:** Comparison with the state-of-the-art antennas at the sub-millimeter wave range.

Ref.	Technique	$\mathrm{Dimensions}\;\mathrm{in}\;{\mathrm{mm}}^{3}(\lambda_{g}^{3})$	Dielectric Constant	Bandwidth (GHz)	Gain (dBi) Min.–Max.
[11]	Array structure	28.823 × 60 × 0.787 (3.1 $\lambda_{g}^{3}$)	2.2	24.6–28.5	8–11.2
[15]	HRI metamaterial	30.5 × 30 × 0.508 (0.99 $\lambda_{g}^{3}$)	2.2	24.5–27.5	5.8–7.4
[17]	High order mode DRA	20 × 20 × 20 (3.56 $\lambda_{g}^{3}$)	2.2	14.1–16.5	9–10.4
[16]	Metamaterial	40 × 24 × 1.6 (13.88 $\lambda_{g}^{3}$)	4.4	24.8–34.52	5–9.53
[19]	Stacked radiator	13 × 11.25 × 1.787 (0.85 $\lambda_{g}^{3}$)	2.2	5G–multiband	N.M–7.6
[20]	DGS	30 × 35 × 0.76 (4.3 $\lambda_{g}^{3}$)	3.66	25.5–29.6	N.M–8.3
[18]	Exciting of quasi-TM 310	26.416 × 26.416 × 0.508 (0.14 $\lambda_{g}^{3}$)	2.2	14.93–15.13	N.M–10.74
This work	SIC Power splitter and ENZ metamaterial	8.5 × 8.5 × 5.5 (2.5 $\lambda_{g}^{3}$)	3.6	28.5–30.5	10–11

## Data Availability

Not applicable.
